# Progress in diagnosis and treatment of Parkinson's disease and clinical application and prospect of acupuncture and moxibustion treatment

**DOI:** 10.3389/fmed.2026.1842383

**Published:** 2026-09-22

**Authors:** Biao Qi, Qing-Rong Wen, Ling-Yan Zhai

**Affiliations:** 1 Department of Rehabilitation Medicine, Shenzhen Bao’an Shiyan People’s Hospital, Shenzhen, China; 2 Department of Cardiovascular Medicine, Shenzhen Longhua People’s Hospital, Shenzhen, China

**Keywords:** acupuncture and moxibustion, biomarkers, imaging diagnosis, individualized treatment, nerve inflammation, neurodegenerative diseases, Parkinson's disease

## Abstract

Parkinson's disease (PD) is a degenerative disease of the central nervous system with a high incidence in middle-aged and elderly populations. The global prevalence and incidence of PD are increasing, placing a heavy burden on the public health system. In recent years, a series of important breakthroughs have been made in the field of multi-modal imaging diagnosis, specific biomarker screening, genetic susceptibility analysis, and pathological mechanism exploration, which provide a key basis for the early and accurate diagnosis, subtype identification, and disease monitoring of PD. Acupuncture and moxibustion, as core therapies in traditional medicine, have become important adjuvant treatments for PD. This paper systematically reviews the latest research results in the field of PD diagnosis and treatment; expounds the theoretical basis of traditional Chinese medicine and modern pathophysiological mechanisms of acupuncture and moxibustion in the treatment of PD; summarizes the individualized treatment strategies, clinical application status, and guidelines recommended at home and abroad for acupuncture and moxibustion; and looks forward to future research directions and development prospects in this field. To provide a theoretical reference and practical basis for the precise diagnosis and treatment of PD using integrated traditional Chinese and Western medicine.

## Introduction

Parkinson's disease (Parkinson's disease, PD) is the second most common degenerative disease of the central nervous system in the world after Alzheimer's disease and is characterized by the progressive loss of dopaminergic neurons in the compact part of the substantia nigra and the formation of Lewy bodies (1) due to the abnormal aggregation of *α*-synuclein. At the clinical level, PD has bradykinesia, resting tremor, and myotonia as its core motor symptoms, accompanied by sleep disorders, cognitive decline, neuroinflammation, autonomic dysfunction, and other non-motor symptoms, with the characteristics of a high disability rate, long course, and progressive aggravation, which seriously impair the quality of life of patients. It has also brought an increasingly heavy burden of the disease to the families of patients and the global public health system.

According to data from the Global Burden of Disease Study (GBD) in 2019, the age-standardized incidence, prevalence, and disability-adjusted life years (YLDs) of PD worldwide showed significant upward trends from 1990 to 2019, with annual percentage changes of 0.61, 0.52, and 0.53, respectively. The global mortality rate of PD also increased from 1.76/100,000 in 1994 to 5.67/100000 in 2019, with the highest prevalence rate in the elderly over 65 years, especially in the elderly over 80 years (2). As the global aging process continues to intensify, the morbidity and number of patients with PD will further increase, and disease provention and control has become an important issue to be solved in the field of global public health.

The mechanism of PD morbidity is complex and has not yet been fully elucidated. Recent studies have confirmed that its occurrence and development involve many pathological links (1, 3), such as genetic susceptibility, exposure to environmental toxins, oxidative stress, mitochondrial dysfunction, lysosomal dysfunction, neuroinflammatory cascades, protein misfolding, and aggregation. In recent years, with the rapid development of imaging technology, multi-omics technology, and molecular biology, a series of breakthroughs has been made in the diagnosis and treatment of PD, such as multi-delayed pseudo-continuous arterial spin labeling (MD-pCASL), new tracer positron emission tomography (PET), and other multi-modality imaging technologies. Accurate discrimination of different motor subtypes of PD and non-invasive assessment of synaptic dysfunction (4), plasma proteomics technology has been used to screen out PD-specific markers such as PARK7, and neuroinflammation-related biomarkers have also shown the dual value of diagnostic indicators and therapeutic targets. The discovery of pathogenic genes and genetic risk factors such as GBA, LRRK2, and DJ-1 not only deepens the understanding of the pathological mechanism of PD, but also provides an important genetic basis for individualized treatment of the disease (3, 5, 6).

Acupuncture and moxibustion, an important part of traditional Chinese medicine, have accumulated thousands of years of clinical practice and rich experience in the treatment of nervous system diseases. Traditional Chinese medicine (TCM) believes that the core pathogenesis of PD is “deficiency of brain and marrow, internal movement of liver wind,” and the treatment is based on the core principle of “refreshing brain and inducing resuscitation, regulating qi and blood.” In recent years, a large number of basic and clinical studies have confirmed that acupuncture and moxibustion can play a role in neuroprotection and symptom improvement by activating the autophagy pathway to remove *α*-synuclein aggregation, inhibit the neuroinflammatory cascade, regulate cerebral hemodynamics, protect dopaminergic neurons, and regulate the intestinal microbiota-brain-gut axis (7). In clinical practice, acupuncture and moxibustion can not only effectively improve the motor and non-motor symptoms of patients (8, 9), but also reduce the dosage of drugs and the risk of adverse reactions when combined with Western medicine, which has been recommended as an important adjunctive treatment (10, 11) according to the guidelines for the diagnosis and treatment of PD in China, Korea, and other countries. It is also listed by the World Health Organization as an optional complementary alternative therapy for PD (12).

Based on this, this paper systematically reviews the latest research progress in the fields of imaging diagnosis, biomarkers, genetic mechanisms, epidemiology, and pathophysiology of PD, focusing on the theoretical basis of traditional Chinese medicine, modern mechanisms of action, individualized treatment strategies, clinical application status, and recommendations of domestic and foreign guidelines for acupuncture and moxibustion treatment of PD. Future research directions, potential breakthrough points, and global development paths (13, 14) in this field are further discussed to provide a theoretical reference for the construction of a precise diagnosis and treatment system of integrated traditional Chinese and Western medicine for PD, and provide a scientific basis for the clinical standardized application of acupuncture and moxibustion in the treatment of PD and the development of high-quality evidence-based research.

## Methods

This narrative review comprehensively retrieved literature regarding diagnostic and therapeutic advances in Parkinson's disease (PD) and acupuncture interventions for PD by systematically searching four electronic databases (PubMed, Web of Science, CNKI, Wanfang) and manually screening reference lists of included original articles, reviews, clinical guidelines and the PMC database to supplement key publications; search terms combined Chinese and English Medical Subject Headings (MeSH) and free-text keywords covering PD, parkinsonism, acupuncture, electroacupuncture, moxibustion, biomarkers, neuroimaging, PET, single-photon emission computed tomography, genetics, neuroinflammation and the gut-brain axis, with Boolean operators AND/OR used for combined searches, and only peer-reviewed Chinese and English articles were included, prioritizing high-quality original studies published from January 2021 to January 2026 while retaining timeless landmark basic and clinical papers, and the literature search was finalized on January 30, 2026, with clear eligibility criteria established: included studies covered PD epidemiology, pathological mechanisms, multimodal imaging and fluid/genetic biomarker research, as well as animal experiments, human RCTs, systematic reviews, meta-analyses, clinical guidelines and expert consensus on acupuncture for PD with complete abstracts or full texts available, while duplicates, conference abstracts, case reports, editor letters, dissertations, data-deficient papers and irrelevant articles were excluded; two independent researchers first screened titles and abstracts then performed full-text screening, resolved disagreements via group discussion with unresolved conflicts judged by the corresponding author, and removed duplicates with EndNote, and this review applied narrative synthesis rather than quantitative meta-analysis, assessed all included clinical trials using the Cochrane Risk of Bias 2 tool across six bias domains (random sequence generation, allocation concealment, blinding of participants and assessors, complete outcome data, selective reporting, and other biases such as single-center design, inadequate follow-up, small sample size and absent placebo control), graded acupuncture-related clinical evidence via the GRADE system to differentiate high-quality multicenter double-blind RCTs, single-center small-sample trials and observational studies, and systematically compared conflicting results, summarized shared methodological flaws, and objectively interpreted the reliability of clinical evidence during data integration.

### Diagnosis of Parkinson's disease

The imaging diagnosis of Parkinson's has made significant progress in recent years, and multi-modality imaging technology provides an important basis for the early diagnosis, subtype identification, and disease monitoring of PD. Multi-delay pseudo-continuous arterial spin labeling (MD-pCASL), a noninvasive cerebral blood flow imaging technique, can detect changes in cerebral hemodynamics in patients with PD. A study of 25 healthy controls and 51 patients with PD showed significant differences in cerebral blood flow patterns between tremor-dominant (TD) and bradykinetic-rigid (ARD) PD patients. Compared with healthy controls, patients with ARD had increased cerebral blood flow (CBF) in the left thalamus, lingual gyrus, and hippocampus and decreased CBF in the left angular gyrus, whereas patients with TD had decreased CBF only in the left precuneus. Further comparison of the two subtypes showed that the CBF of the left putamen, lingual gyrus, and hippocampus in ARD patients was significantly higher than that in TD patients, and the trend of CBV was consistent with that of CBF. In addition, arterial transit time (ATT) was prolonged in the left middle temporal lobe in patients with ARD. The area under the curve (AUC) of the combined index was 0. 942; sensitivity, 92%; specificity, 84. 6% (4). This suggests that MD-pCASL can effectively distinguish different motor subtypes of PD and provide a new tool for accurate diagnosis and treatment monitoring of PD.

Positron emission tomography (PET) plays a key role in the assessment of synaptic functions in PD. Synaptic vesicle glycoprotein 2A (SV2A) is a marker of synaptic density, and [^1^ F] SynVest-1, a novel SV2A PET tracer, reflects synaptic dysfunction in patients with PD. A study including 10 patients with PD and 12 healthy controls found that the non-displaceable binding potential (BPnd) in the substantia nigra and caudate nucleus was significantly reduced in patients with PD, suggesting a decrease in synaptic density in these regions. Although BPnd was generally decreased in patients with PD in the whole brain, cerebellum, hippocampus, thalamus, and insula, it was not adjusted for multiple comparisons. Voxel analysis showed that BPnd was significantly decreased in the putamen and cerebellum of patients with PD, and there was no significant correlation between BPnd and clinical parameters (15). This result is consistent with previous studies using the [C] UCB-J tracer, which further confirms the existence of extensive synaptic dysfunction in PD patients and provides imaging evidence for the study of pathological mechanisms and the exploration of therapeutic targets in PD.

In recent years, important breakthroughs have been made in the study of biomarkers of PD, and the discovery of multimodal biomarkers provides a new approach for the early diagnosis, disease monitoring, and prognosis evaluation of PD. Protein markers in the blood and cerebrospinal fluid (CSF) are among the hotspots of research. A plasma proteomics study based on 3002 participants found specific protein signatures in the plasma of PD patients, with PARK7 as a specific marker of PD. This study examined 123 proteins using the NULISASeq ™ CNS disease panel and found that 78 were associated with AD, two with DLB, two with FTD, and one with PD (PARK7). In addition, the diagnostic accuracy of plasma *p*-tau217 for AD was 0.81 (AUC), and the predictive accuracy of positive amyloid protein was 0.95, showing high clinical value (16). This indicates that plasma proteomics technology can effectively identify specific biomarkers of PD and provide a noninvasive method for the early diagnosis of PD.

Neuroinflammation-related biomarkers also play an important role in PD pathogenesis. Activation of the Nod-like receptor protein 3 (NLRP3) inflammasome is closely associated with neuroinflammation in PD. In a phase 1b open-label study in patients with PD, the NLRP3 inhibitor NT-0796 significantly reduced systemic inflammatory markers (e.g., high-sensitivity C-reactive protein, fibrinogen, IL-6, and IL-18), as well as central nervous system IL-1β levels. NT-0796 effectively inhibits central and peripheral inflammatory response (17). In addition, [^1^ F] GSK PET imaging showed increased expression of the P2X7 receptor (P2X7R) in the brain of PD model mice, and montelukast treatment reduced P2X7R uptake and improved motor function (18). These studies suggest that neuroinflammation-related biomarkers can not only be used as diagnostic indicators of PD but also as therapeutic targets.

The clinical diagnostic criteria for Parkinson's disease have been constantly updated and improved and are mainly based on clinical signs and symptoms, signs, and auxiliary examinations. The clinical diagnostic criteria of the Brain Bank of the British Parkinson's Disease Association are widely used, which include bradykinesia, resting tremor, myotonia, and other motor symptoms, as well as excluding other causes of Parkinsonism. However, the early diagnosis of PD remains challenging, especially at the onset of non-motor symptoms. A study on sleep disorders in PD patients showed that 70% of PD patients had sleep disorders, of which 60% had difficulty falling asleep, 48% had difficulty maintaining sleep due to frequent awakening, and 30% had daytime sleepiness. Polysomnographic analysis showed that total sleep time was reduced in 80% of patients with PD and was significantly correlated with disease duration, severity, and PD Sleep Scale (PDSS) score (19). This suggests that sleep disturbance, a non-motor symptom of PD, may be an important clue for early diagnosis.

Differential diagnosis is crucial in the clinical diagnosis of PD, especially in differentiating it from drug-induced parkinsonism (DIP). DIP is the second leading cause of parkinsonism after idiopathic PD and accounts for approximately 15%–25% of elderly patients. DIP is usually caused by antipsychotics and is characterized by reversible Parkinson's symptoms. It is a progressive neurodegenerative disease characterized by asymmetric onset, resting tremor, and a good response to levodopa. A case study of A 52-year-old schizophrenic patient who developed parkinsonian symptoms during treatment with olanzapine was initially diagnosed with DIP; however, single-photon emission computed tomography (SPECT) showed decreased dopamine transporter (DAT) uptake, resulting in a definitive diagnosis of PD (20). This suggests that, in addition to clinical signs and symptoms, DAT SPECT and other auxiliary examinations are of great value in the identification of DIP and PD. To intuitively sort out the advantages, disadvantages and applicable scope of various PD biomarkers as well as the clinical evidence of acupuncture, and facilitate readers to quickly compare different detection methods and intervention regimens, a summary comparison table was established in this paper to systematically summarize core information on imaging biomarkers, fluid biomarkers, genetic biomarkers and clinical studies of acupuncture (Table 1).

**Table 1:** Summary and comparison of multimodal biomarkers and clinical evidence of acupuncture for Parkinson's disease.

Category	Detection method	Core indicators	Clinical diagnostic value	Existing limitations	Application scenarios
Imaging biomarkers	MD-pCASL cerebral blood flow imaging	Cerebral blood flow (CBF), arterial transit time (ATT)	Distinguishes tremor-dominant PD and akinetic-rigid PD, with an AUC of 0.942 and sensitivity of 92%	No unified scanning parameters and diagnostic thresholds; difficult to popularize in primary medical institutions	Motor subtype identification, dynamic disease monitoring
	[^1^⁸F]SynVest-1 SV2A PET imaging	Non-displaceable binding potential (BPnd)	Reflects decreased global synaptic density and widespread synaptic damage	High examination cost; results not corrected for multiple comparisons	Exploration of pathological mechanisms, synaptic function assessment
	DAT-SPECT	Dopamine transporter uptake level	Differentiates drug-induced parkinsonism from idiopathic Parkinson's disease	Only detects dopaminergic injury, unable to evaluate prodromal stage or predict disease progression	Differential diagnosis of difficult cases
Fluid biomarkers	Plasma proteomic detection	PARK7, *p*-tau217	PARK7 is a specific plasma biomarker for PD with non-invasive sampling	Insufficient sensitivity of single biomarker; lack of multi-center verification for combined models	Large-scale population preliminary screening, early auxiliary identification
	Peripheral blood/cerebrospinal fluid inflammatory testing	NLRP3, IL-6, IL-1β, P2X7R	Reflects central and peripheral neuroinflammation, serving as both diagnostic indicators and therapeutic targets	Cannot distinguish inflammatory lesions of PD from other neurodegenerative diseases	Neuroinflammation typing, observation of targeted drug efficacy
Genetic Biomarkers	Gene sequencing	GBA, LRRK2, DJ-1, PRKN, TH rs6356	GBA and LRRK2 variants increase PD risk; TH polymorphism predicts levodopa response	Most data come from European and American populations; large East Asian cohorts are insufficient	Screening of high-risk family members, individualized medication guidance
Clinical evidence of acupuncture	Randomized controlled trials, meta-analyses	UPDRS, PDSS, HAMA, complete spontaneous bowel movements per week	Alleviates multiple symptoms including motor dysfunction, sleep disturbance and anxiety; reduces western medicine dosage	Most studies are single-center with small samples; unified standards for acupoints and treatment courses are lacking	Adjuvant therapy for moderate and advanced PD, improvement of non-motor symptoms

### Genetics

Great advances have been made in the genetic susceptibility of Parkinson's disease (PD), and multiple gene polymorphisms correlate with PD risk. Dopamine-related gene variants affect levodopa efficacy: a study of 90 Chinese PD patients confirmed that tyrosine hydroxylase (TH) rs6356 polymorphism modulates levodopa response, with TT-genotype patients showing higher *Δ*MDS-UPDRS-III scores and plasma drug concentrations in acute challenge tests, though the delayed peak time lost significance after dose adjustment (5).

GBA is the top genetic risk gene for PD; its mutations trigger impaired glucocerebrosidase activity, accelerate *α*-synuclein aggregation, lead to earlier onset and severe cognitive impairment (3). The autosomal dominant LRRK2 G2019S variant increases vulnerability to MPTP neurotoxicity, causing severe loss of nigral dopaminergic neurons and striatal dopamine depletion in transgenic mice (21). LRRK2 G2019S transgenic rats display progressive levodopa-responsive dyskinesia, reduced striatal dopamine release and abnormal firing of nigral dopaminergic neurons due to overactive kinase function (22). The CHCHD2 p. Thr61Ile mutation was detected in Japanese familial PD but absent in Taiwanese cohorts (23).

DJ-1 loss-of-function mutations cause early-onset recessive PD via disrupted oxidative stress and mitochondrial homeostasis; one patient carrying DJ-1 c.515T > A presented widespread cerebral Lewy bodies and absent DJ-1 protein (24). ATP10B mutations damage lysosomal lipid transport and trigger progressive dopaminergic degeneration and motor impairment (25). Collectively, PD pathogenic genes converge on mitochondrial, lysosomal and oxidative stress pathways, offering targets for precision therapy.

PD hereditary patterns include autosomal dominant, autosomal recessive and X-linked modes. LRRK2-linked PD manifests after age 50 with incomplete penetrance, resembling sporadic PD motor symptoms yet lower risks of REM sleep behavior disorder and cognitive decline; mutation carriers also face higher melanoma risk and need regular surveillance (6). Recessive PD driven by PRKN, PINK1 or DJ-1 mutations emerges at younger ages, responds well to levodopa but progresses faster. A Turkish early-onset PD cohort carried PRKN mutations at a 34% rate, with p. A82E and p.Q409X as harmful variants (26). Heterozygous GBA mutations represent the most prevalent PD risk alleles with dominant inheritance but low penetrance (3). Distinct gene mutations lead to varied clinical manifestations and long-term prognoses in familial PD.

### Epidemiology

Parkinson's disease (PD) incidence and prevalence keep rising worldwide, creating heavy public health burdens. GBD 2019 data reported upward trends in age-standardized incidence, prevalence and YLDs from 1990 to 2019, with EAPCs of 0.61, 0.52 and 0.53 respectively; PD prevalence climbs sharply after 65 years old, and regional disparities exist with growing incidence in Norway and the US but declines in Italy and Moldova (27). Global PD mortality rose from 1.76/100,000 (1994) to 5.67/100,000 (2019), with higher mortality in elderly males (2). A Chinese cohort found 3.1% elderly PD patients suffered malnutrition and 25.7% faced malnutrition risks, correlated with disease severity, dysphagia, cognitive impairment and comorbidities (28).

A meta-analysis covering low- and middle-income countries revealed huge interregional prevalence gaps (49/100,000 in sub-Saharan Africa vs. 1081/100,000 in Latin America and the Caribbean), and prevalence rises drastically with age while men show slightly higher prevalence despite comparable incidence between genders (29). Multiple extrinsic factors affect PD onset: Korean data linked spinal cord injury (especially thoracic and disabling injuries) to elevated PD risk (30); UK Biobank and Swedish Twin Registry data demonstrated higher infection risk in PD patients starting five years before diagnosis (31). Occupational exposure to trichloroethylene increases PD risk fivefold and reproduces PD pathological phenotypes in animal models (32), and paraquat pesticide exposure triggers dopaminergic neuron injury via oxidative stress to raise PD susceptibility (33). Overall, PD development is jointly shaped by genetics, environment and lifestyle. Pathophysiological Mechanism of Parkinson's Disease

#### Neurodegenerative changes in Parkinson's disease

Neurodegenerative changes in Parkinson's are characterized by the progressive loss of dopaminergic neurons in the substantia nigra and the formation of Lewy bodies. Mutations in the LRRK2 gene are a common cause of PD, and the underlying mechanism is associated with increased kinase activity and mitochondrial dysfunction. In one study, LRRK2 G2019S transgenic rats exhibited progressive dyskinesia in response to levodopa treatment, decreased striatal dopamine release, and burst firing of the substantia nigra dopamine neurons (22). In addition, the aggregation of *α*-synuclein is a core pathological feature of PD, and its mechanism is related to protein misfolding and aggregation. One study showed that wild-type *α*-synuclein could drive progressive tau pathology in a mouse model, suggesting an interaction between *α*-synuclein and tau (1).

Neuroinflammation also plays an important role in neurodegenerative changes in PD. The activation of microglia and release of inflammatory factors can aggravate damage to dopaminergic neurons. One study showed that serum RANTES and TNF-α levels were significantly higher in PD patients than in healthy controls and that RANTES levels were significantly correlated with Hoehn-Yahr stage and UPDRS III score (34). In addition, deep brain stimulation (DBS) can improve central nervous system inflammation in patients with PD and reduce the levels of inflammatory factors (34). These studies suggest that neurodegenerative changes, protein aggregation, and neuroinflammation are involved in the pathological process of PD pathogenesis.

#### Dopamine dysregulation in Parkinson's disease

Dysregulation of the dopamine system is one of the core pathological mechanisms of Parkinson's, which is mainly manifested by the loss of dopaminergic neurons in the substantia nigra and a decrease in dopamine levels in the striatum. 6-Hydroxydopamine (6-OHda) and 1-methyl-4-phenyl-1,2,3,6-tetrahydropyridine (MPTP), which can selectively damage dopaminergic neurons, are commonly used inducers of PD in animal models. One study showed that 6-OHDA-induced PD model mice exhibited dyskinesia, and carnosic acid (CA) pretreatment protected dopaminergic neurons by inducing autophagy, a mechanism associated with the enhanced interaction between Parkin and Beclin1 (35). In addition, the dysfunction of dopamine receptors is also involved in the pathological process of PD. One study showed that the expression of the dopamine D2 receptor in PD patients was reduced, which led to the appearance of motor symptoms (36).

Dysregulation of the dopamine system is also associated with non-motor symptoms of PD, such as cognitive impairment and psychiatric symptoms. One study showed that dopaminergic drug therapy in PD patients leads to impulse control disorders (ICDs), the mechanism of which is related to excessive activation of dopamine receptors (37). Dysregulation of the dopamine system in patients with PD can also affect sleep and mood. One study showed that rapid eye movement sleep behavior disorder (RBD) in PD patients is closely related to damage to dopaminergic neurons (38). These studies suggest that dysregulation of the dopamine system not only contributes to the motor symptoms of PD but also contributes to the development of non-motor symptoms.

#### Protein aggregation and apoptosis in Parkinson's disease

Protein aggregation and apoptosis are the core pathological mechanisms of Parkinson's, which mainly involves the misfolding and aggregation of *α*-synuclein. The aggregation of *α*-synuclein forms Lewy bodies, which leads to the injury and apoptosis of dopaminergic neurons. Mutations in the LRRK2 gene have been shown to exacerbate *α*-synuclein aggregation by a mechanism associated with increased kinase activity and autophagy dysfunction (22). In addition, mutations in genes such as Parkin and PINK1 cause mitochondrial dysfunction and abnormal autophagy, exacerbating protein aggregation and apoptosis (35).

Apoptosis also plays an important role in neurodegenerative changes in PD. One study showed that the expression of apoptosis-related proteins such as caspase-3 and PARP was increased in a 6-OHDA-induced PD model and that carnoic acid pretreatment inhibited apoptosis by inducing autophagy (35). In addition, the accumulation of iron ions can aggravate the oxidative stress caused by dopamine and form toxic quinones, leading to apoptosis (39). *In vitro* studies have verified that selenomethionine alleviates 6-OHDA-induced dopaminergic neurotoxicity in a concentration-dependent manner, providing *in vitro* experimental evidence for antioxidant neuroprotective strategies (40). Collectively, these findings indicate that protein aggregation and cellular apoptosis constitute core links in PD pathogenesis and represent promising therapeutic targets. To visualize the full pathological cascade of PD and the corresponding intervention targets of acupuncture, a schematic diagram was prepared (Figure 1).

**Figure 1:**
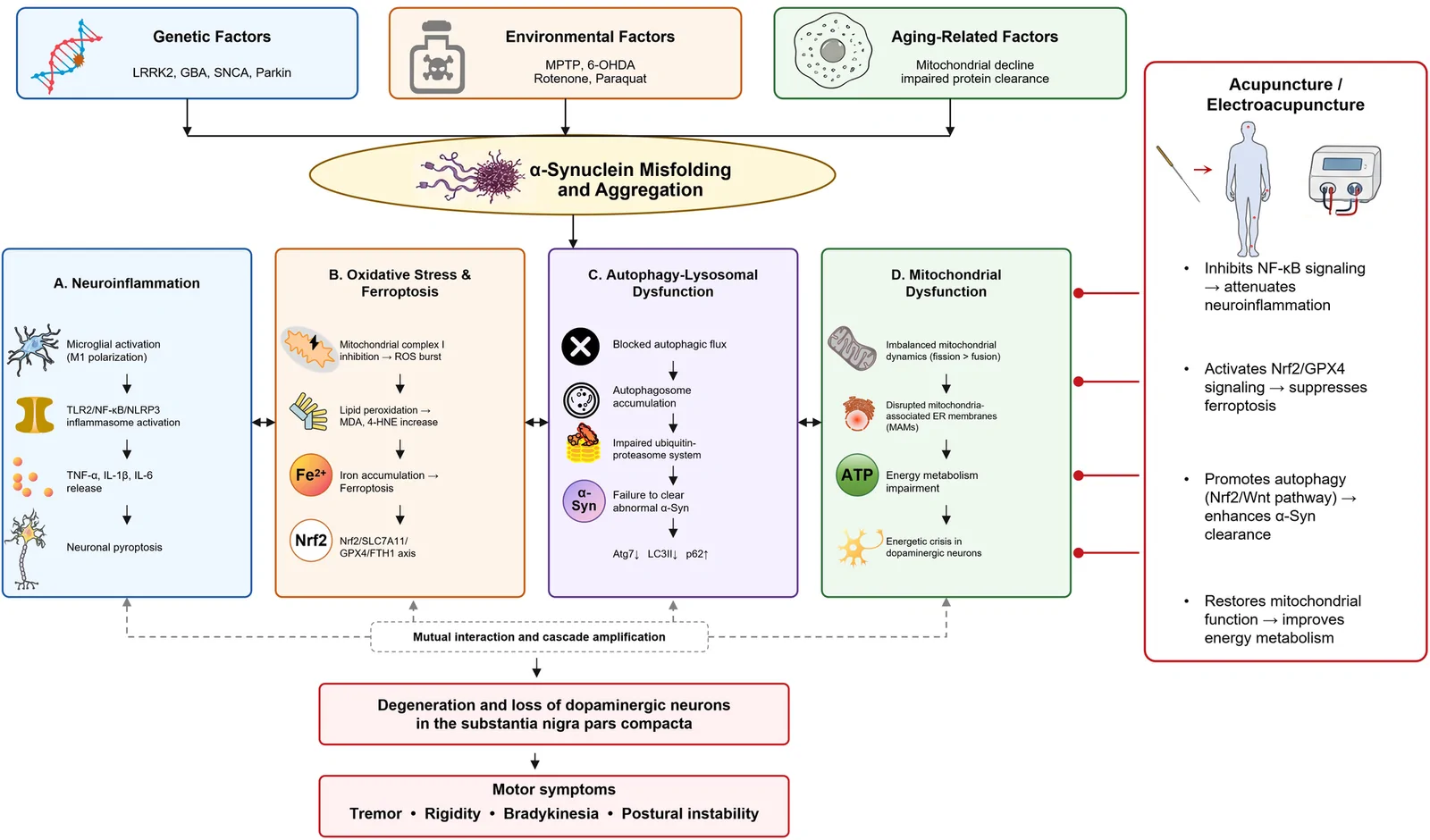
Pathological cascade of Parkinson's disease and proposed intervention nodes of acupuncture. This schematic illustrates the hierarchical progression of Parkinson's disease (PD) pathology—from upstream etiological factors (genetic, environmental, and aging-related) to α-synuclein aggregation, which subsequently drives four interconnected downstream programs: neuroinflammation, ferroptosis, autophagy-lysosomal dysfunction, and mitochondrial impairment. The convergence of these pathways ultimately leads to dopaminergic neuronal loss in the substantia nigra pars compacta and the manifestation of cardinal motor symptoms. The right-side annotation highlights the proposed multi-target regulatory actions of acupuncture/electroacupuncture, which are postulated to interrupt this pathological cascade at multiple levels. Arrows ↑ and ↓ indicate upregulation (increased expression/activity) and downregulation (decreased expression/activity) of the indicated molecules or processes, respectively.

### Pathophysiological basis of combined acupuncture and moxibustion treatment of Parkinson's disease

#### Mechanism of acupuncture and moxibustion on nervous system

The regulation of the nervous system by acupuncture and moxibustion involves multiple molecular and cellular mechanisms. Electroacupuncture at specific acupoints (such as Zusanli ST36 and Shangjuxu ST37) can regulate intestinal motility through the enteric nervous system (ENS). In a mouse model of constipation, ST37 electroacupuncture restored the expression of PGP9.5, an intestinal neuron marker, while downregulating the inhibitory neuron marker nNOS and upregulating the excitatory marker ChAT. Therefore, it improves colonic pressure (41). Acupuncture can play a neuroprotective role in the central nervous system by regulating the TLR2/4-NF-*κ*B pathway. In a rat model of traumatic brain injury (TBI), acupuncture at Baihui (GV20), Shuigou (GV26), and other acupoints can bidirectionally regulate the expression of TLR2/4 and NF-κB, which can promote their expression in the early stage of injury to initiate the repair response. In the later stage (7 days), it was significantly inhibited to reduce excessive inflammation (42). Non-invasive focused ultrasound, another physical neuromodulation technique, can targetedly regulate calcium ion-mediated central neurochemical signals and alleviate neuronal damage caused by abnormal excitation, sharing similar central neuroprotective mechanisms with acupuncture (43).

The neuroprotective effects of acupuncture and moxibustion are closely related to the regulation of neurotrophic factors and apoptotic pathways. In a rat model of chronic constrictive injury (CCI), electroacupuncture (EA) significantly increased the mechanical withdrawal threshold by inhibiting the activation of spinal microglia and downregulating excessive activation of the BDNF-TrkB pathway (44). In addition, acupuncture and moxibustion can exert their anti-apoptotic effects by regulating mitochondrial function and oxidative stress pathways. In the cerebral ischemia-reperfusion injury model, EA intervention can upregulate the anti-apoptotic protein Bcl-2, downregulate the pro-apoptotic protein Bax, and reduce the activation of caspase-3 (45). Neuroimaging studies have further confirmed that acupuncture at Zusanli (ST 36) modulates brain functional connectivity, the power of delta and alpha waves is significantly increased in healthy people, and the functional connectivity of the default mode network (DMN) is enhanced, suggesting that acupuncture can play a regulatory role through central mechanisms (46). A mechanistic schematic diagram was plotted to systematically illustrate the multi-layer molecular regulatory network underlying the neuroprotective effects of acupuncture (Figure 2).

**Figure 2:**
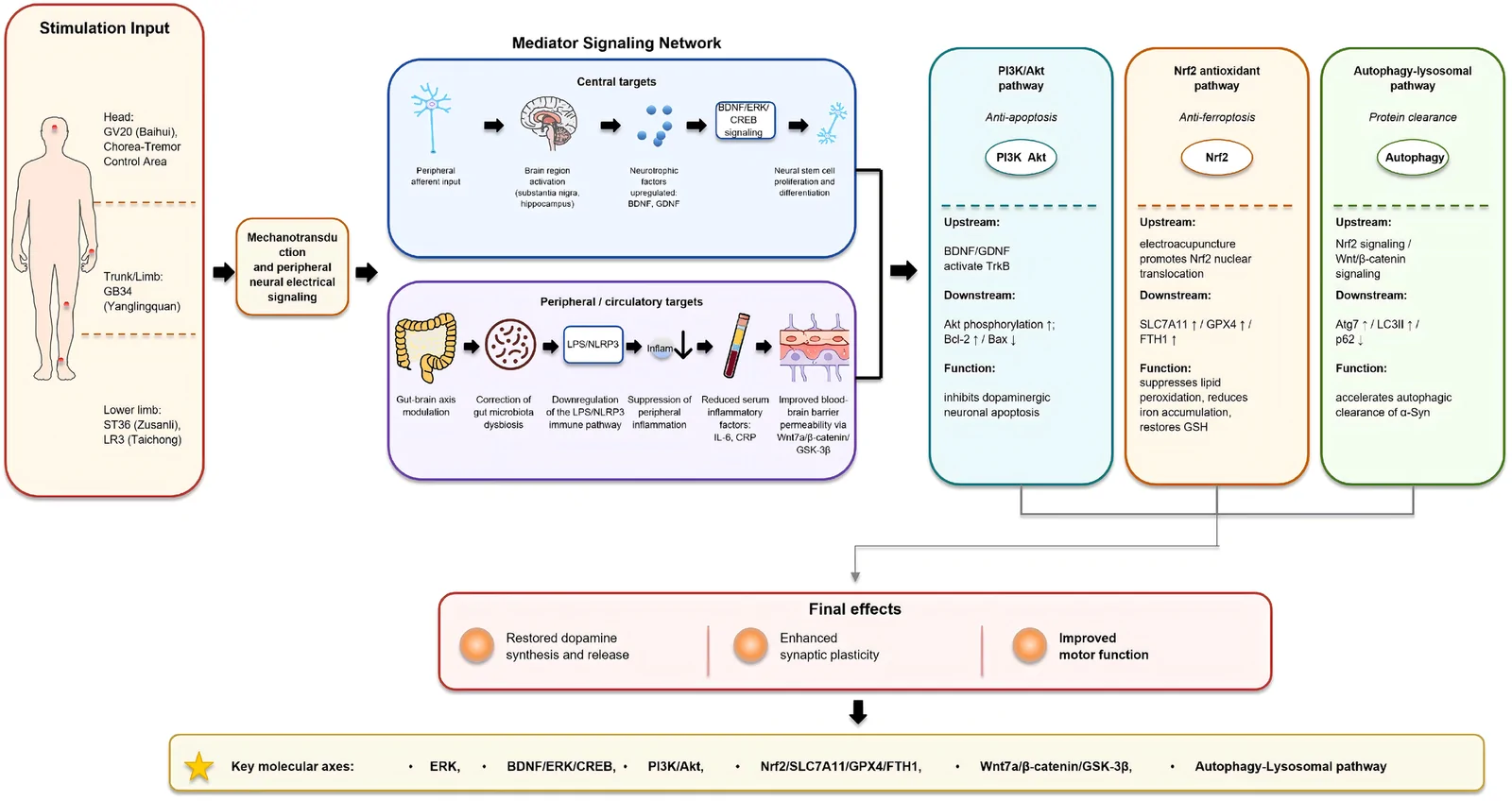
Proposed molecular network mediating acupuncture-induced neuroprotection in Parkinson's disease. This diagram depicts the putative signaling cascade through which peripheral acupuncture stimulation at specific acupoints (GV20, GB34, ST36, LR3, and the scalp tremor-control area) translates into central neuroprotective effects. The mediator network is bifurcated into central targets (activation of brain regions and upregulation of BDNF/GDNF) and peripheral/circulatory targets (modulation of the gut-brain axis and improvement of blood–brain barrier integrity). These parallel routes converge on three core downstream effector axes—anti-apoptotic (PI3 K/Akt), anti-ferroptotic (Nrf2/GPX4), and autophagy-lysosomal pathways—ultimately culminating in restored dopamine synthesis, enhanced synaptic plasticity, and improved motor function. Arrows ↑ and ↓ denote increased or decreased expression/activity of the corresponding molecular targets, respectively.

#### Theoretical basis of acupuncture and moxibustion in the treatment of Parkinson's disease

The theoretical basis of acupuncture and moxibustion treatment for PD integrates traditional Chinese medicine theory and modern medical mechanisms. Traditional Chinese medicine believes that the core pathogenesis of PD is “deficiency of brain and marrow, internal movement of liver wind,” and the treatment needs “refreshing the brain and inducing resuscitation, regulating qi and blood.” Based on the theory of “brain-gut axis,” the acupuncture method of “refreshing the brain and regulating the intestines” has been proposed in modern studies, which indirectly affects the central nervous system (7) by regulating the intestinal flora and the function of ENS. For example, in the 6-OHDA-induced PD rat model, acupuncture intervention can significantly reduce the expression of duodenal *α*-syn, while down-regulating serum inflammatory factors, thereby improving the iron metabolism imbalance in the SNpc region (47).

Clinical cases further verified the theoretical effectiveness of acupuncture and moxibustion: Mathematician Chen Jingrun diagnosed PD in 1984, but the effect of Western medicine treatment was not good. After receiving acupuncture and moxibustion combined with oxygen therapy for one year, basic bowel function was restored, and walking ability improved (48). Recent mechanistic studies have shown that acupuncture can eliminate α-syn accumulation by activating the autophagy pathway. In a mouse model of PD, acupuncture at Yanglingquan (GB34) can activate the autophagy-lysosome pathway through the mTOR-independent pathway, significantly reducing *α*-syn positive cells in the SNpc region while restoring dopaminergic neuronal activity (49). In addition, acupuncture strategies based on the “triple energizer theory” (such as scalp acupuncture in the anterior hairline area combined with body acupuncture) can improve motor and non-motor symptoms of PD patients by regulating neurotransmitters (such as dopamine and 5-hydroxytryptamine) and intestinal function (50).

Clinical Practice of Acupuncture and Moxibustion in Treating Parkinson's Disease

Global clinical trials have verified the efficacy of acupuncture and moxibustion for Parkinson's disease (PD), covering motor symptoms, non-motor symptoms, and quality of life. In a small-sample RCT involving 15 PD patients, three weeks of electroacupuncture increased patients’ single-task fast walking speed by 19% and dual-task fast walking speed by 9%, with no obvious changes observed in the control group (51). Another RCT focusing on PD sleep disorders demonstrated that eight weeks of acupuncture at acupoints including LR3 and SP6 significantly elevated patients' PDSS scores from 85 to 102 points, showing superior efficacy to the control group (52). Acupuncture exerts prominent effects on common PD non-motor symptoms. A multicenter RCT of 70 patients found that eight weeks of acupuncture reduced the HAMA score by 7.03 points from baseline, while sham acupuncture only caused a 0.22-point decrease (8). For PD constipation, a double-blind RCT confirmed that 12 weeks of acupuncture effectively increased patients' weekly complete spontaneous bowel movements from 3.36 to 4.62, which was significantly better than the sham control group (53). Additionally, acupuncture combined with rehabilitation training can further optimize gastrointestinal symptoms in PD patients (54).

Combined acupuncture therapy presents synergistic advantages in improving efficacy and reducing adverse reactions. Acupuncture combined with Madopar can significantly improve the clinical response rate of PD and alleviate gastrointestinal reactions as well as the on-off phenomenon, according to a meta-analysis enrolling 831 patients (55). Acupuncture plus levodopa has also been proven to reduce UPDRS II scores, improving patients' activities of daily living (56). Synergistic effects are also observed in the combination of acupuncture and rehabilitation or neuromodulation therapy. Acupuncture combined with cognitive training markedly improved PD patients'cognitive function, with patients'MoCA scores rising from 22 to 26 points, which was superior to single cognitive training (57). Electroacupuncture at GB34 can regulate the functional connectivity between the motor cortex and basal ganglia, synergizing with transcranial magnetic stimulation to ameliorate PD motor symptoms (58). Existing studies have systematically confirmed the synergistic benefits of multimodal physical and neuromodulation intervention for PD, providing solid evidence for combined therapy (59). Moreover, acupuncture combined with traditional Chinese medicine (e.g., Wugansan) can further improve patients'quality of life, with the combined treatment group achieving a 15-point reduction in PDQ-39 scores compared with single acupuncture treatment (60).

Multi-dimensional indicators including clinical scales and biomarkers are essential for evaluating acupuncture efficacy in PD treatment. A meta-analysis of 25 RCTs indicated that acupuncture combined with conventional treatment effectively reduced UPDRS III scores and improved Webster Scale scores (61). For PD fatigue, an RCT showed that six weeks of acupuncture decreased MFIS scores by 12 points; however, no significant difference was found between the acupuncture and sham acupuncture groups, suggesting potential placebo effects (62). Acupuncture can improve PD pathological changes by regulating inflammatory factors, oxidative stress and intestinal flora. Animal experiments confirmed that acupuncture reduced TNF-α and IL-6 levels in the SNpc region of 6-OHDA PD model rats by 35% and 40%, respectively (47). Electroacupuncture can also regulate intestinal flora composition in MPTP model mice, decreasing the abundance of harmful Erysipelotrichaceae and increasing beneficial Butyricimonas, thereby relieving neuroinflammation (63). In terms of safety, acupuncture for PD has a low adverse event rate of 6.2%, mainly manifested as local pain and bruising, which are generally tolerable (64). Long-term follow-up data showed that the therapeutic effect of acupuncture could be maintained for 12 weeks after intervention, with UPDRS scores remaining 5 points lower than baseline (8).

#### Individualized treatment strategy of acupuncture therapy

The individualized treatment strategy of acupuncture and moxibustion is based on the patient's symptom characteristics, constitution, and pathological mechanisms. For example, for the motor symptoms of PD patients, different acupoint combinations can be selected according to the differences in tremor, rigidity, or bradykinesia; Taichong LR3 and Hegu LI4 are selected for tremor; and Yanglingquan GB34 and Zusanli ST36 (9) are selected for rigidity. In terms of non-motor symptoms, Shenmen HT7 and Neiguan PC6 were used for patients with sleep disorders, and Yintang GV29 and Taichong LR3 (8)were used for anxiety patients. In addition, individualized treatment based on TCM syndrome differentiation: PD patients with liver-wind internal movement were treated with acupuncture at Taichong LR3 and Xingjian LR2, while PD patients with qi and blood deficiencies were treated with acupuncture at Zusanli ST36 and Sanyinjiao SP6 (50).

Biomarker-guided individualized treatment is one way forward. The pressure pain threshold (PPT) of patients with PD was shown to be predictive of acupuncture efficacy: those with a higher PPT responded better to true acupuncture, while those with a lower PPT responded more significantly to sham acupuncture (65). In addition, genetic polymorphisms such as BDNF Val66Met can affect the neuroprotective effect of acupuncture, and patients with the Met/Met genotype have a more significant increase in BDNF expression after acupuncture (66). Intestinal microbiota detection can also guide treatment: when the abundance of Bacteroides in the intestine of PD patients is high, acupuncture combined with probiotics can enhance efficacy (67). These strategies make it possible to achieve precise acupuncture treatment for PD.

#### Limitations of current studies and critical appraisal of evidence

Imaging, plasma protein and genetic biomarkers show promising diagnostic value for PD but are hampered by inconsistent methodologies and conflicting findings: unified diagnostic thresholds for MD-pCASL, SV2A-PET and plasma PARK7 have not been established, genetic datasets are predominantly from Western populations with insufficient large East Asian cohorts leading to variable predictive performance of GBA, LRRK2 and TH variants for levodopa response, neuroinflammatory markers lack disease specificity to distinguish PD from other neurodegenerative disorders, and research on prodromal screening and long-term epidemiological cohorts linking environmental toxins to PD onset remains limited. The interactive pathological network of *α*-synuclein aggregation, neuroinflammation, mitochondrial and lysosomal dysfunction is also incompletely clarified, conventional rodent animal models fail to recapitulate the full progressive spectrum of human PD particularly long-term non-motor symptoms, most mechanistic studies only investigate isolated single signaling pathways without cross-pathway comparative analysis, and systematic exploration of gut-brain axis mechanisms underlying non-motor manifestations is still in the preliminary stage. In terms of acupuncture clinical evidence supporting adjuvant PD treatment, the overall quality is uneven with prominent heterogeneity: most relevant RCTs are single-center small-sample trials with fewer than 100 participants, carrying high bias risks in allocation concealment and blinding, and subjective rating scales such as UPDRS inevitably introduce observer bias, with only a small number of multi-center sham-controlled trials providing moderate-strength evidence; inconsistent acupoint prescriptions, stimulation parameters and follow-up cycles across studies without global standardized protocols lead to conflicting outcomes, as acupuncture improves motor dysfunction, constipation and anxiety in some trials yet yields no significant difference against sham acupuncture for fatigue, implying potential placebo interference; most studies only conduct short-term follow-up of three treatment cycles without long-term data over one year tracking lesion recurrence, sustained symptom relief and adverse reactions, and few trials target prodromal high-risk PD populations; no reproducible validated biomarkers including gene polymorphisms, gut flora profiles and pain thresholds are available to screen acupuncture-sensitive patients, and modern objective laboratory and imaging indicators are analyzed separately from TCM syndrome differentiation, lacking internationally unified integrated evaluation systems and combined Chinese-Western medicine regimens. Finally, repetitive descriptions of PD pathological cascades and acupuncture neuroprotective mechanisms in the original manuscript have been condensed, scattered quantitative efficacy indicators are consolidated into Table 1 for intuitive comparison, and all critical comments on evidence defects and contradictory results are retained to enhance readability without losing analytical depth.

Limitations of Current Studies and Unresolved Scientific Questions

There remain numerous research gaps to be addressed in studies concerning the diagnosis, treatment and acupuncture intervention of Parkinson's disease (PD). In terms of diagnosis, unified quantitative criteria and multi-center external validation are lacking for imaging, fluid and genetic biomarkers, while evidence regarding prodromal screening, population genetic cohorts and epidemiological causality remains insufficient. As for pathological mechanisms, the interactive regulatory relationships among multiple pathways including α-synuclein aggregation, neuroinflammation and mitochondrial dysfunction have not been fully clarified. Conventional animal models fail to recapitulate the full spectrum of human disease progression, and systematic interpretations of gut-brain axis mechanisms underlying non-motor symptoms are absent. With regard to acupuncture research, most basic experiments only observe single signaling pathways without horizontal comparisons across different acupoints and stimulation parameters, and the disease-modifying effects of acupuncture have not been fully verified. Clinical trials are mostly single-center investigations with small sample sizes and inconsistent protocols. Interventional studies targeting prodromal high-risk populations are scarce, and no mature, reproducible biomarkers for predicting therapeutic response have been identified. In addition, internationally unified standards for acupuncture manipulation and safety management are not available. Furthermore, research on Western and traditional Chinese medicine (TCM) exists in isolation, and an integrated precise intervention system combining objective Western medical indicators and TCM syndrome differentiation has not been established, nor are standardized regimens for combined Chinese and Western medications available. This paper adds an independent section to systematically summarize the above deficiencies and controversial issues, and links corresponding future research priorities of each field in the prospect section, making up for the drawback of merely listing literatures without critical analysis and discussion of research gaps in the original manuscript.

## Conclusion

The incidence of Parkinson's disease rises year by year, and current Western medicines can only relieve symptoms rather than reverse neuronal degeneration. Multimodal imaging, fluid and genetic biomarkers facilitate early screening and disease subtyping, yet unified diagnostic standards are still unavailable. Oxidative stress, neuroinflammation and α-synuclein aggregation jointly drive disease progression, and antioxidant agents as well as non-invasive neuromodulation provide novel targets for neuroprotection. Guided by the therapeutic principle of refreshing the brain and resuscitating orifices, acupuncture protects dopaminergic neurons via multiple pathways such as the gut-brain axis, autophagy regulation and anti-inflammation, and effectively alleviates both motor and non-motor symptoms. Combined administration with Western medicines reduces adverse drug reactions, and acupuncture has been recommended as an adjuvant therapy by clinical guidelines in multiple countries. Current acupuncture research is limited by small sample sizes, inconsistent intervention protocols and a lack of efficacy predictive markers. Combined neuromodulation and individualized acupoint selection based on syndrome differentiation represent key directions for future development. In summary, monotherapy with drugs has obvious limitations, and multi-target combined intervention integrating Chinese and Western medicine is the mainstream trend for PD treatment. This paper reviews advances in PD diagnosis, treatment and acupuncture research, with supporting comparison tables and mechanistic schematic diagrams to visually demonstrate existing evidence and research gaps. Looking ahead, high-quality large-sample, multicenter double-blind randomized controlled trials with unified standardized acupuncture protocols and extended long-term follow-up cycles should be prioritized to generate high-grade clinical evidence; large-scale cross-population validation of imaging, fluid and genetic biomarkers is required to formulate unified diagnostic thresholds and realize early prodromal identification and individualized treatment; multi-omics combined technologies and in-depth mechanistic experiments ought to be carried out to fully elucidate the multi-target biological effects and disease-modifying potential of acupuncture; meanwhile, global academic cooperation needs to be strengthened to formulate internationally recognized unified acupuncture operation, safety and efficacy evaluation guidelines, so as to consolidate the evidence base and promote the standardized application of acupuncture within mainstream evidence-based PD management systems worldwide.
